# Management of Bilateral Mandibular Fused Teeth

**DOI:** 10.7759/cureus.7899

**Published:** 2020-04-30

**Authors:** Victor Goh, Oscar D Tse

**Affiliations:** 1 Periodontology, The National University of Malaysia, Kuala Lumpur, MYS; 2 Dentistry, Hong Kong Sanatorium and Hospital, Hong Kong, HKG

**Keywords:** fused teeth, bone regeneration, periodontics, oral health

## Abstract

Tooth fusion is a developmental dental anomaly that may affect both the deciduous and permanent dentition. Such anomalies may cause problems such as caries, periodontal disease or even esthetic impairments, which will require intervention. In the present case, a young patient was referred to the periodontal clinic for management of bilateral mandibular fused teeth as part of the orthodontic treatment. On the lower right, the teeth involved were incompletely fused involving only the cervical region. On the lower left, the two teeth were completely fused from the crown to the apex. A surgical resection was carried out on the fused teeth on the right, while the fused teeth on the left was undisturbed. Orthodontic treatment was later carried out to align both the upper and lower arch. The patient was satisfied with treatment outcome.

## Introduction

Tooth fusion is defined as the union between two or more separate developing teeth. Such anomaly may be complete or incomplete depending on the developmental stage of the associated tooth buds [1]. If contact between two tooth buds occurs before calcification, complete fusion ensues. This may be seen clinically as an abnormally wide crown usually with a groove that separates the mesial and distal moiety [1]. If contact occurs after crown formation, incomplete fusion occurs at the root level. Incomplete fused teeth may present with separate pulp chambers and root canals [1-2].

The etiology of fusion remains unclear and may be associated with genetics, racial predisposition, or even trauma during tooth development [1, 3]. Fusion almost always occurs in the anterior region, involving the lateral incisors and canines [1, 3-4]. Although fused teeth may not bring about any clinical problems, treatment is needed when these anomalies cause caries, periodontal disease, crowding, and esthetic impairments [5].

Management of fused teeth usually requires a multidisciplinary approach. Surgical, endodontic, orthodontic, and restorative interventions are usually proposed in combination to manage fused teeth. However, each case must be considered separately taking into account the patient’s wishes and needs. The purpose of this case report is to illustrate the different treatment approaches employed for managing bilateral (complete vs. incomplete) fused mandibular teeth.

## Case presentation

A 19-year-old male patient was referred to the periodontal clinic for surgical management of fused teeth on his lower left and right quadrants. On his left, 32 was fused to 33; on the right, 42 was fused to 43.

**Figure 1:**
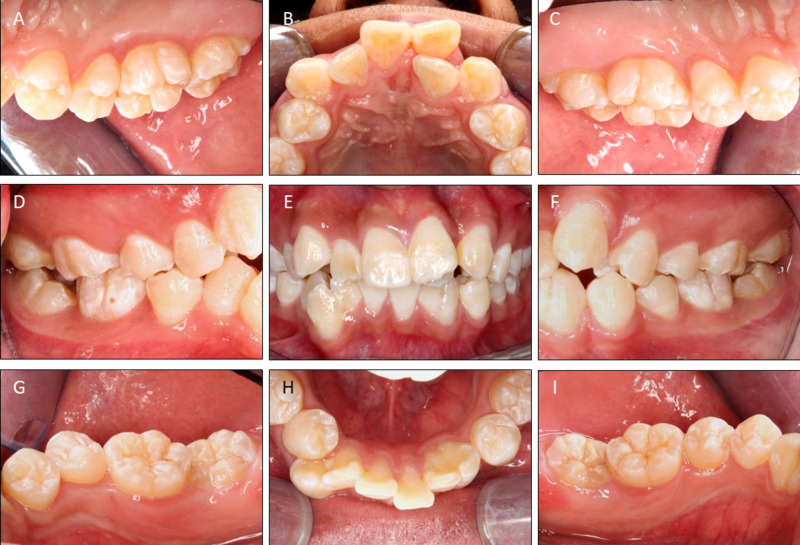
Pre-operative intraoral photographs. Note the fused 42-43 and 32-33.

The patient was systematically healthy and was first seen in the orthodontic clinic for consultation. He presented with class I skeletal pattern, and crowding on both arches. His 22 was palatally displaced and there was an upper midline shift to the left. The midline on the lower arch was slightly shifted to the right. The fused 32-33 was relatively well aligned within the lower arch, but the fused 42-43 was slightly disto-buccally rotated with the 43 buccally displaced (Figure 1E).

**Figure 2:**
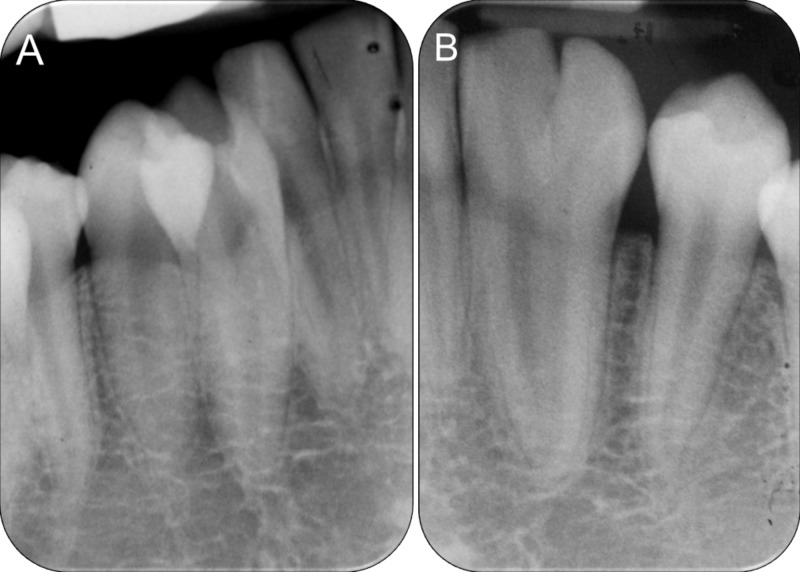
Pre-operative periapical radiographs. (A) Fused 42-43 with separate pulp chambers, (B) Fused 32-33 with merged pulp chambers at the apical third.

Radiographic examination showed that 42-43 were incompletely fused only at the cervical region with two separate roots and individual pulp chambers all the way to the apex (Figure 2A). Contralaterally, 32-33 was completely fused down to the apex with one root and two seemingly “separate” pulp chambers merging at the apical third (Figure 2B).

After discussion, it was decided to keep the 32-33 as it was, with the patient’s consent that this may compromise the final esthetics. As for 42-43, a surgical resection between the two teeth with extraction of the 42-moeity was decided. 42 was selected for extraction as 43 had more periodontal support and removal of 42 would provide enough space to correct the malocclusion on the lower arch. Ridge preservation with bone graft (BioOss® from Geistlich®, Wolhusen, Switzerland) [6] and collagen membrane (Osteobiol® from Tecnoss®, Pianezza TO, Italy) [7] were planned to maintain the dimensions of the 42 area while the teeth were being moved.

**Figure 3:**
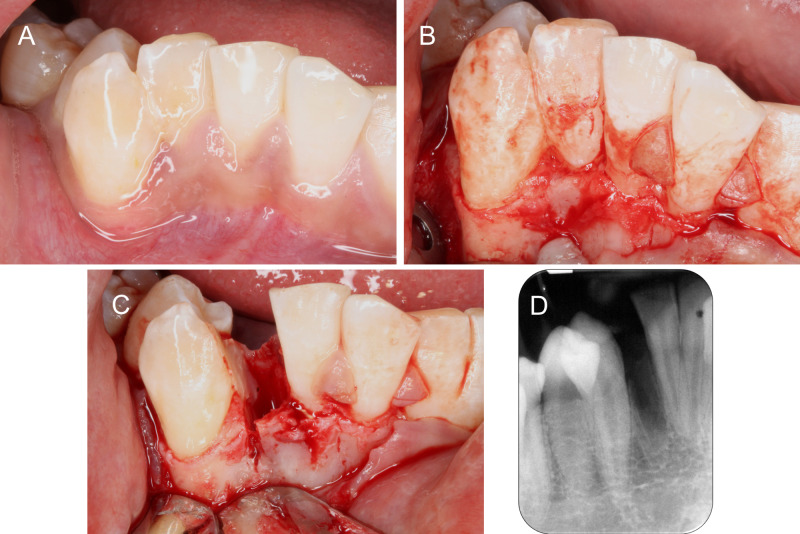
Resection of 42-43. (A) Pre-operative condition of 42-43. (B) Full flap exposure to visualize the site of fusion. (C) 42 sectioned and extracted. (D) Periapical radiograph confirming complete removal of 42 and thorough debridement of 43.

On the day of surgery, a full thickness buccal envelope flap was raised from 31-distal to 43-distal. 42-43 was debrided to fully expose the area of fusion which ended at the cervical region (Figure 3B). This was carefully sectioned with a fine tapered diamond bur. Drilling was directed towards the 42 to preserve as much tooth structure on the 43 as possible, and also maintain the thin inter-septal bone between the 42 and 43 (Figure 3C). After atraumatic extraction of the 42 with periotomes and forcep, the rough edges on 43 were smoothened with Gracey’s curettes. A postextraction radiograph was taken to confirm that the mesial surface of 43 was thoroughly smoothened and no remnant of 42 was left (Figure 3D).

**Figure 4:**
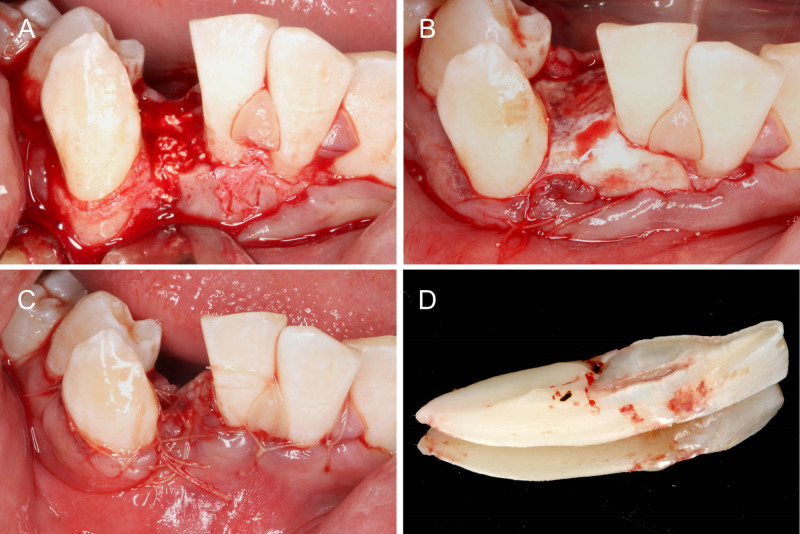
Ridge preservation. (A) Socket filled with bone graft. (B) Grafted area covered with a collagen membrane. (C) Primary wound closure. (D) Site of fusion on 42 which extends to the cervical area only.

The socket on 42 was filled with bone graft (Figure 4A) and covered completely with a collagen membrane (Figure 4B). The wound was primarily closed with resorbable sutures (Figure 4C). The patient was prescribed ibuprofen 400 mg and amoxycillin 500 mg three times a day for five days. He was advised not to brush the surgical site and to rinse with chlorhexidine mouthwash two to three times daily.

Sutures were removed two weeks postsurgery and the area healed uneventfully. Tooth sensibility testing with cold and electric pulp test was carried out at one- and four months postsurgery. Results were positive. Orthodontic treatment commenced four months after surgical treatment.

**Figure 5:**
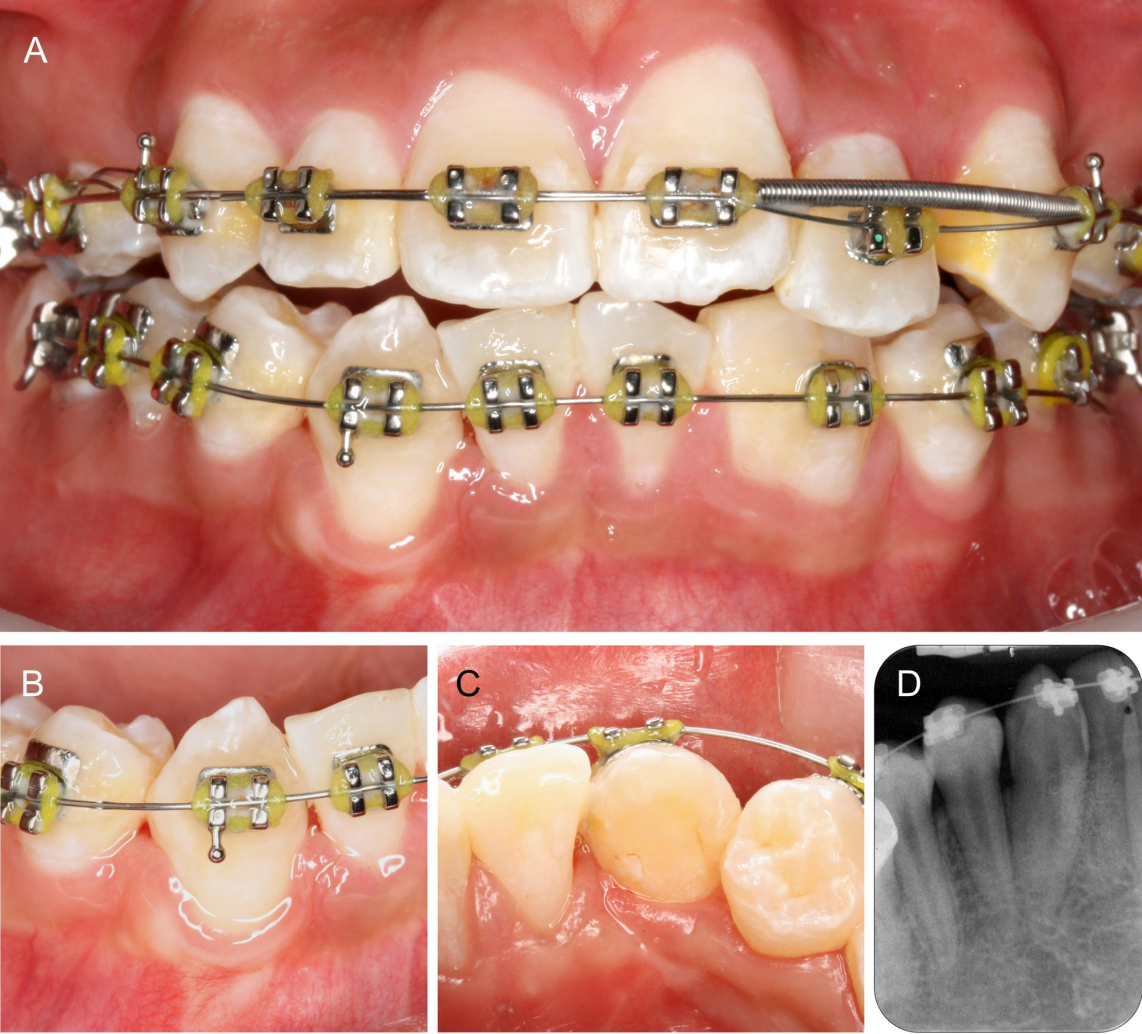
Twelve months follow-up. (A) Space closure of the lower arch at 12 months. (B-C) Good contact between 43 and the adjacent teeth both buccal and lingually. (D) Periapical radiograph of 43 with no abnormalities.

The patient was reviewed every six months after orthodontic treatment started. Complete closure of the 42-space was achieved 12 months after orthodontic treatment (Figure 5A-C). 43 remained responsive to cold and electric pulp test throughout treatment. Periapical radiograph of 43 showed normal periodontal space surrounded by bone and some remnant bone graft (Figure 5D).

**Figure 6:**
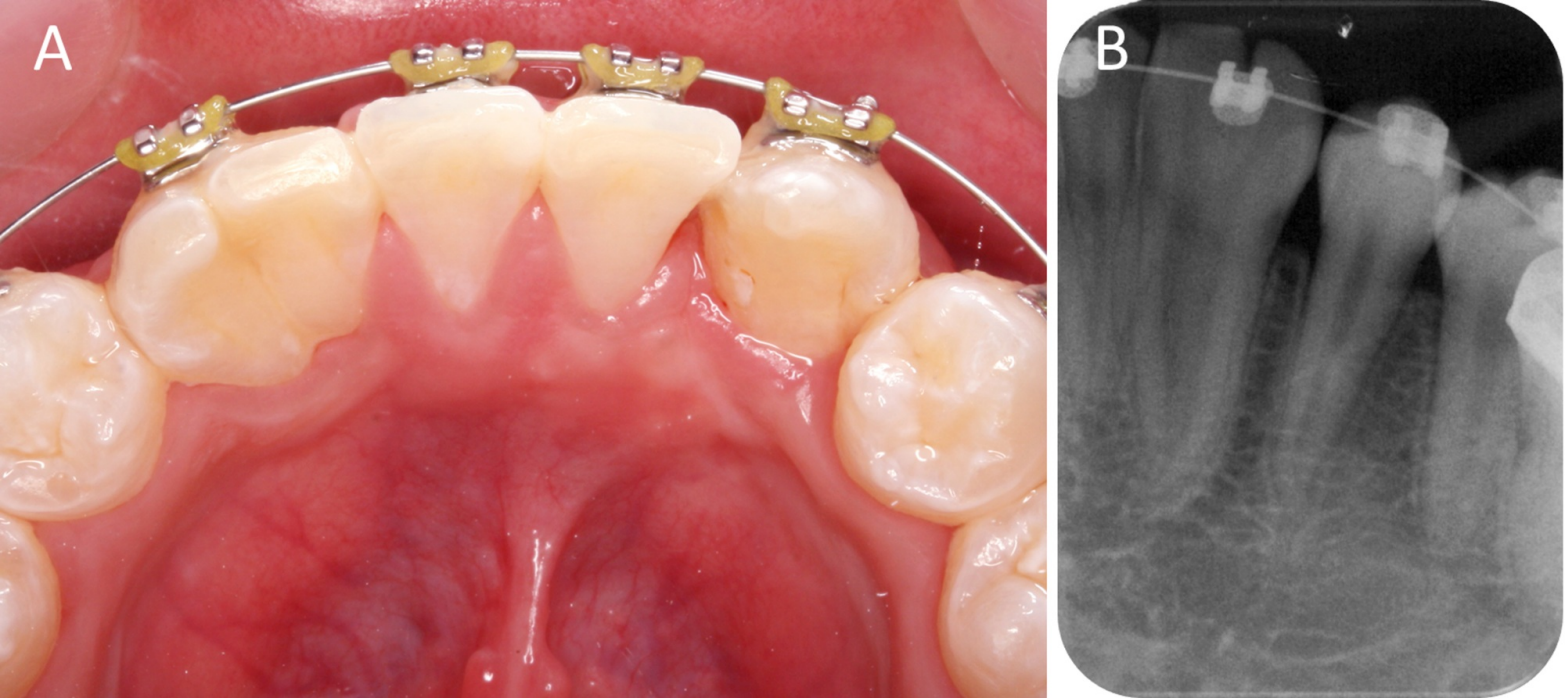
Fused 32-33 aligned within the arch.

The fused 32-33 was moved to improve the midline (Figure 6A). No other treatment for 32-33 was planned as the patient was not too concerned about the esthetics of his lower arch. The patient continued with orthodontic treatment and will be viewed periodically.

## Discussion

In the case presented, the aim of the therapy was to create space on the lower jaw through separation and extraction of one of the fused teeth and correct the arch discrepancy through orthodontic treatment.

Tooth extraction without bone grafting results in significant reduction in vertical and horizontal bone dimensions [8]. The utilization of a bone grafting material for alveolar ridge preservation reduces the resorption process occurring after tooth extraction [9]. Although such measures to maintain the ridge dimensions remain debatable, it was deemed necessary as the original buccal-lingual dimension of the 42 was quite narrow, with a nonuniform buccal plate thickness of <1 mm. Moreover, surgical trauma during sectioning of the buccal plate was unavoidable to allow separation of the 42 moiety (Figure 3C). Slight ridge expansion during extraction and grafting of the socket was carried out to preserve the alveolar bone dimensions, while waiting for the 43 to be moved into the edentulous site. This enhances pre-orthodontic conditions, allowing teeth to be moved with reduced chances of dehiscence and root resorption [10]. Orthodontic movement into grafted sites does not appear to have any detrimental effect on the tooth nor does it affect tooth movement [10]. Ridge preservation was carried out in this case to the desired effect and the larger 43 was moved into the preserved extraction space within 12 months without any complications. The alveolar ridge dimensions were adequately maintained and 43 was surrounded by a healthy band of keratinized tissue.

There are various treatment options for fused teeth in the permanent dentition and the treatment of choice is determined by the condition of the teeth involved and each patient’s particular needs [4]. In most cases, surgical division with or without endodontic therapy may be performed [2, 4-5]. In the present case, 42-43 was selected for surgical treatment as the space requirements to correct the malocclusion on the lower arch was minimal. Extraction in the right quadrant alone was sufficient. Furthermore, as 42-43 was fused only at the cervical region with two separate root canals, the risk of endodontic complications was assessed by an endodontist and deemed minimal. This was opposed to the contralateral site where 32-33 were completely fused. If more space was required to move teeth towards the lower left, a more aggressive treatment option for 32-33 may be considered. One option may be to extract both 32-33 and redistribute the space for a dental implant after orthodontics. Dental implants in a young patient are not without their long-term complications and retention of natural teeth is desired [11]. Alternatively, an extraoral hemisection and immediate replantation of either 32 or 33, with subsequent root canal therapy may be considered [12]. Fortunately, this was not the case and 32-33 was undisturbed. If esthetics were a concern, the fused 32-33 may later be prepared for a composite or porcelain veneer to mask the fusion [13].

Careful clinical examination aided by clear, high definition radiographs is crucial for optimal treatment planning. Currently, intraoral radiography is the imaging technique of choice for the management of endodontic cases [14]. Conventional intraoral periapical radiographs provide a high definition image at a low radiation dose. In recent years, cone beam computed tomography (CBCT) is frequently used for complex endodontic diagnosis and management of complications [15]. However, current CBCT images have lower resolution than conventional intraoral radiographs [14] and patients are subjected to higher radiation dose and financial cost [14-15]. The periapical radiographs used to plan and monitor the present case shows the whole image of the roots especially 42-43, and was considered adequate after discussion with the endodontist in charge [4]. The ridge appeared narrow upon clinical examination and a CBCT would confirm such findings but have no added value to the overall treatment plan. No invasive treatment was planned for 32-33 and any additional radiation exposure was unnecessary. 

## Conclusions

There are many treatment modalities to manage fused teeth. Selective resection of fused teeth with subsequent orthodontic alignment appears to be an acceptable treatment option in certain cases. Although the evidence on ridge preservation procedures to facilitate orthodontic tooth movement is lacking, such measures appear to have no detrimental effect on orthodontic tooth movement and may be employed in selected cases. When patients are young, retention of their natural dentition via a more conservative approach with minimal surgical intervention may be preferred. For such treatment to be successful, treatment planning and consensus between different dental specialties are needed.
